# Food Colour Additives: A Synoptical Overview on Their Chemical Properties, Applications in Food Products, and Health Side Effects

**DOI:** 10.3390/foods11030379

**Published:** 2022-01-28

**Authors:** Maria Manuela Silva, Fernando Henrique Reboredo, Fernando Cebola Lidon

**Affiliations:** 1 ESEAG/Grupo Universidade Lusófona, 1749-024 Lisboa, Portugal; 2 GeoBioTec Research Center, Faculdade de Ciências e Tecnologia, Campus da Caparica, Universidade Nova de Lisboa, 2829-516 Caparica, Portugal; fhr@fct.unl.pt (F.H.R.); fjl@fct.unl.pt (F.C.L.)

**Keywords:** dyes, food colour additives, naturally derived colourants, side effects of food colours, synthetic colourants

## Abstract

Colour is one of the most relevant organoleptic attributes that directly affects consumers’ acceptance and food selection. However, as food colouring pigments are generally unstable and become modified during processing, in order to maintain or restore product colour uniformity, colourants are added to food products around the world. In this context, although they are still widely used, synthetic food colorants, due to their potential hazards, are being replaced by those obtained from natural origins. Indeed, numerous side effects and toxicities, at both the medium and long-terms—namely allergic reactions, and behavioral and neurocognitive effects—have been related to the use of synthetic colourants, whereas their naturally-derived counterparts seem to provide a somewhat high-quality and effective contribution as a health promoter. In order to further understand the implications of the use of synthetic and naturally derived food colourants, this review aims to provide a synoptical approach to the chemical characteristics, properties, uses and side effects on health of those which are currently allowed and applied during food processing.

## 1. Introduction

Food properties, namely colours, which are a visual feature associated with the spectral distribution of light resulting from the interaction with matter, largely determine consumer’s satisfaction and expectations, affecting their choice and eating desires. Food colours affect recognition and product acceptability (warning consumers against eating spoiled food which is hazardous to health), as they are a beam of sensory perceptions such as sight, smell, and taste [1]. Still, the degradation of existing natural pigments can prevail during food processing, which in most cases requires addition of colourants to restore or enhance colour. Thus, a large number of food products incorporate colours in order to obtain a pleasing appearance or dye feature. For instance, in order to enhance consumers preferences, food colours are usually applied to edible ices, desserts, pastry and fine bakery products, decorations and the coatings of pastry, confectionary products, sauces, fruit juices, snacks and soft drinks, and alcoholic beverages [1,2,3].

In the European Union, the use of food colours—similarly to the use of other food additives—is regulated by specific laws, with these being the reported categories of food products, maximum usable quantities, chemical characterization and purity [3,4,5,6,7,8,9,10]. Accordingly, colourants can only be used if they are integrated into three categories: having a defined acceptable daily intake (i.e., previously determined and allowed for use), or being permitted to be used only in special or specific cases. Besides this, each food additive has a code in the EU, which includes the letter E (for Europe), followed by three or four digits. The numbering scheme follows that of the International Numbering System (INS), as determined by the Codex Alimentarius Committee.

Food colours can be synthetic, synthesized equally to the natural, or naturally derived. Although most of the natural food colours are derived from plants, some others are obtained from animals or even ores [2]. Most natural colorants have some disadvantages, such as reactivity towards the other ingredients of foodstuffs or in the presence of aromas/odours, as well as instability in water, or when exposed to light and heat. Moreover, contrarily to synthetic dyes, the consumption of natural food colourants can have significant benefits; they are in demand for their reliability, functionality, biological potential and health benefits. Natural colours are obtained from nature, and can be isolated by more or less complicated extraction processes, whereas synthetic dyes are chemically synthesised. However, sometimes it is not easy to classify them into synthetic or natural colours, as food colour processing can become very complicated, because although they may start with natural substances, a set of chemical processes involving extraction or modification procedures of the initial substance might be required.

The synthetic food colours do not occur in nature due to chemical structures, but have the advantage of prevailing in the form of powders, pastes or granulates, and are soluble in water. Nevertheless, some synthetic colour additives may present health problems, namely allergenic problems, which in children can cause hyperactivity and even mutagenic and/or carcinogenic pathologies [1,2,3,4,5,6,7,8,9,10,11,12,13,14,15,16,17,18,19]. Therefore, some food safety studies have led to the banning of some synthetic food additives, such as E128 (i.e., the Red 2G and E154 Brown FK) [3,7,20,21]. Similarly, the food colouring E160f (Ethyl-β-apo-8’-carotenate (ethyl ester of β-apo-8’-carotenic acid)) is no longer included in the European Union additives list [3,7].

In order to surpass the health side effects of the synthetic dyes, their replacement with natural food colourants is frequently an option. For instance, the use of the red food colouring cochineal (E120), which is prepared from an insect—i.e., the cochineal—was stepped up and the use of the red colour obtained from Beetroot Red (E162) and the green food colour of chlorophylls (E140) was also increased. Still, natural dyes are also a target of food safety studies, with some additives being replaced by others with greater safety guarantees. For instance, recently three additives extracted from annatto—(E160b(i), E160b(ii) and E160(iii))—were replaced by the new additives E160b(i), E160b(ii) [10].

Considering the importance safety limits of colourants use in food processing, this review aims to provide information about their general properties, usage purposes and human health side effects.

## 2. Uses, Characteristics and Side Effects of Food Colourants

### 2.1. Natural Food Colours and Food Colours Synthesized Equally to the Natural

Food, as a basic need for human consumers, must be wholesome and safe. However, in this context, colour is also one of the most impressive attributes of foodstuffs, which directly determines the preference, selection and eating desires of the consumers. Still, the utilization of colouring additives in food is confronted with debate. On a global scale, the use of colours in food has faced challenges with disagreement, particularly when added at high doses (i.e., when exceeding the recommended doses). Nevertheless, at the industrial level, the demand for natural colours for food processing is increasing, mostly due to concerns about health risks triggered by some synthetic additives that can be toxic at metabolic, physiological and toxicological levels. Considering, among others, genotoxic and carcinogenic risks, urticaria, asthma, nausea, eczema, bronchitis, bronchitis, bronchospasm, headache, reduced blood coagulation and hyperactivity, in the European Union only the authorized use of natural food colours and those synthesised equally to the natural are clearly regulated [7].

#### 2.1.1. Curcumin

**Figure 1:**
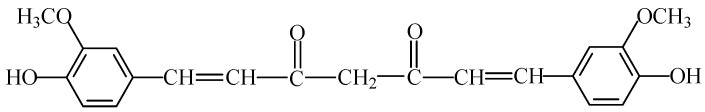
Structural formula of curcumin (E100).

Curcumin (E100), also named as “Cl natural yellow 3”, “yellow–saffron”, “turmeric yellow” or “diferoyl methane” [7] is obtained from the rhizome of *Curcuma longa* L., and furnishes a yellow or orange-yellow colour to food products. This food colour consists essentially of curcumin (Figure 1), two other derivatives at different proportions, and small amounts of naturally occurring oils and resins in the raw material. Curcumin is insoluble in water and diethyl ether, but soluble in ethanol and glacial acetic acid [22].

Curcumin is marketed as a spice under the name of “Indian saffron”, and can be used as a partially purified food colour in the form of powdered preparations of the plant rhizome and oleoresin. In these cases, it also has flavouring properties.

This dye can be applied in most food products, namely mustard, curry, paella, flavoured fermented milk products, flavoured drinks, confectionery including breath refreshing and chewing gum, pastries and fine bakery products, desserts, edible ices, seasoning, fish paste and crustacean paste, precooked crustaceans, smoked fish, sausages, edible cheese rind, processed cheese, salami and pâtés, meal substitutes, soups, dried potato granules and flakes, jams and jellies, marmalades, fat and oil emulsions including spreadable fats, snacks, alcoholic beverages, and other foods in which yellow is desired [3]. 

Curcumin (E100) has a very low toxicity and no genotoxicity [23], and its acceptable daily intake is 15.75 mg/kg_bw_ [24]. Although it has low bioavailability, this colourant can induce some adverse effects [25], but also seems to have anticancer and antioxidant properties [26]. However, at low doses it is apparently an antioxidant, whereas at high ones it enhances the production of reactive oxygen species in cells [27] by inducing topoisomerase II-mediated DNA damage [28,29] and inactivating tumor suppressor protein p53 [30]. Furthermore, although some animal studies showed that curcumin is rapidly metabolised and excreted (mainly via faeces), the data is somewhat contradictory with respect to the absorption of curcumin [31]. It has been also reported that it can cause allergic reactions [16], as well as mutations in bacteria; when fed to pigs, it increases the weight of their thyroid glands causing, in high doses, severe damage [11]. With overdoses of curcumin (i.e., when administrated daily with 7.87 mg curcumin/kg_bw_), El Malky et al [32] further reported elevated serum and a massive aggregation of inflammatory cells in the portal area of the liver of rats. Nevertheless, the use of curcumin as a food colouring agent, in albino rats, does alter humoral immunity [33], reportedly having antioxidant, anti-inflammatory, and antitumor properties [34]. In humans, curcumin ranging between 0.9 and 3.6 g/day—over 1–4 months—promotes nausea, diarrhea and other adverse reactions with increased serum ALP and LDH in humans [35]. Moreover, curcumin can be used at doses higher than 8 g/day if no effective therapies exist [35].

#### 2.1.2. Riboflavin and Riboflavin-5’-Phosphate

**Figure 2:**
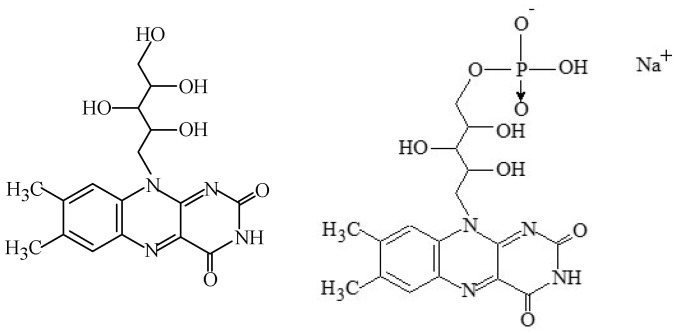
Structural formulae of riboflavin (E101i) and riboflavin-5’-phosphate (E101ii).

Riboflavin, also identified as “lactoflavin” or “vitamin B_2_” (C_17_H_20_N_4_O_6_), and “riboflavin-5’-phosphate” (C_17_H_20_N_4_NaO_9_P·2H_2_O) are water-soluble colourants synthesized by plants and several microorganisms. They are essential micronutrients in the human diet, acting as precursors to flavine adenine dinucleotide and flavin mononucleotide, which function as hydrogen carriers in biological redox processes. These food colours are yellow or orange-yellow crystalline powders, with a slight odour [7,36], with the codes E101(i) and E101(ii), respectively (Figure 2). Although it has been known for many years that bacteria using fermentation technology could produce riboflavin, only recently has a pure product been obtained using a genetically modified strain of *Bacillus subtilis* or the fungus *Ashbya gossypii*. 

Nowadays, riboflavin is obtained by chemical synthesis or biotechnological methods, whereas riboflavin-5’-phosphate is produced by chemical synthesis from riboflavin and phosphoric acid. Milk is the main source of riboflavin, but green vegetables and liver also have appreciable quantities. 

As a food colour, riboflavin gives a yellow colour to whey, and is thermally stable. However, it is sensitive to sunlight and fluorescent light, leading to decomposition reactions that alter the aroma and taste of food products. This effect can be important, for example, in sterilized milk bottled in glass containers.

The acceptable daily intake of riboflavin is 0.5 mg/kg_bw_ [37], but is relatively underused, and when used as a food colour, advertising for the vitamin enrichment of food products cannot be reported. Besides this, the additives E101i and E101ii can be applied in most food products, namely flavoured fermented milk products, edible ices, desserts, edible cheese rind, flavoured drinks, confectionery including breath refreshing and chewing gum, soups, sausages, seasoning, fish paste and crustacean paste, precooked crustaceans, smoked fish, vegetables in vinegar, fruit preserves, dried potato granules and flakes, and brine or oil [3,9].

Although vitamin B_2_ is essential to the human body, its lack does not produce any specific disease. Besides this, no adverse toxic, genotoxic, cytotoxic, or allergic effects have been related to riboflavin. In rats, sodium riboflavin-5′-phosphate is rapidly dephosphorylated to free riboflavin in the intestinal mucosa and then metabolized using specific metabolic pathways [37]. Still, it can affect mouth mucosa, which is not usually serious. Non-severe deficiency states are relatively common, and ingestion in some excess does not appear to be harmful after elimination through urine [16]. Thus, at the currently authorised uses and levels, E101(i) and E101(ii) are unlikely to be of concern as food additives [37].

#### 2.1.3. Cochineal, Carminic Acid or Carmines 

Cochineal, carminic acid or carmines, also named “Cl natural red 4”, (with the chemical formula C_22_H_20_O_13_) is a food colour identified as E120, which is freely soluble in water [38] (FAO, 2000) and shows a red colour [7,39]. 

**Figure 3:**
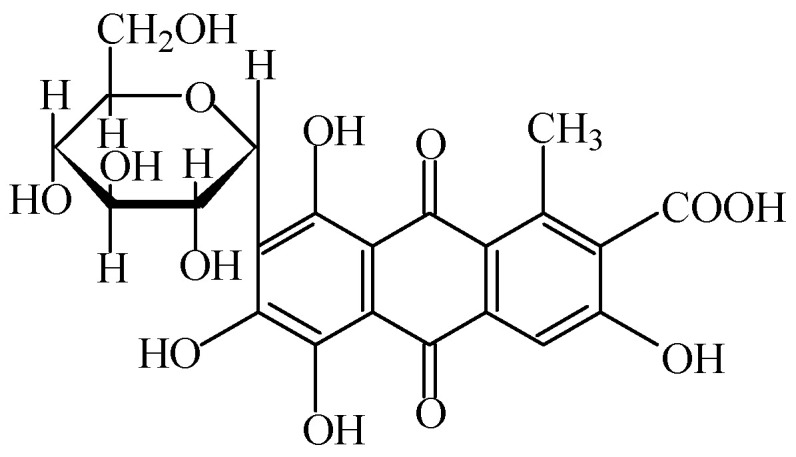
Structural formula of carminic acid (E120).

Carmines and carminic acid (Figure 3) can be obtained from aqueous, aqueous alcoholic or alcoholic extracts of cochineal, and consist of the dried bodies of the female insect *Dactylopius coccus* Costa. The insects of the *Coccidae* family are parasites of some species of cacti. During the last century, the Canary Islands were the main production centre, but today this product can be obtained in large quantities in Peru and other countries of America. The insects are so small that about 100,000 are need to obtain 1 kg of product. However, they are very rich in the food colourant, reaching up to about 20% of their dry weight. The chemical principle of this colourant is the carminic acid, but the substance obtained by extraction with hot water (from insects) alone has no colour. The food colourant itself is obtained through aluminum or calcium’s addition to this extracted product. For some applications, especially beverages, ammonia is added instead of metal [1,2]. Aluminum lakes of carminic acid (carmines) can be formed (i.e., these substances are thought to be present in the molar ratio 1:2). In commercial products, the colouring principle is associated with ammonium, calcium, potassium or sodium cations (singly or combined, with these cations eventually being in excess). Commercial products may also contain proteinaceous material derived from the insect source, and free carminate, or a small residue of unbound aluminum cations [7]. 

Carminic acid is a natural food colour, with a purple or red colour, which can be widely used, namely in preserved red fruits, fruit syrups, ice creams, meat products (such as sausages, chorizo and salami, pâtés, breakfast sausages, and as a meat preparation defined by [40]), lactic products (such as yogurt and fresh flavoured and other processed cheeses), ripened cheese, desserts, edible cheese rinds, flavoured drinks, seasoning, marmalades and jams, pastries and fine bakery, confectionery (including breath refreshing and chewing gum), breakfast cereals flavoured with fruits, fish paste and crustacean paste, precooked crustaceans, smoked fish, some alcoholic beverages, and wine-based snacks [3]. 

Since 2000, the acceptable daily intake for cochineal, carminic acid and carmine (E 120) has been limited to 5 mg/kg_bw_ [41]. The ionisation properties of carminic acid suggest that these compounds can be absorbed into human tissues, but acute, short-term, subchronic, carcinogenicity, reproduction and developmental toxicity studies conducted in rats or mice did not show any toxicological potential [39]. Moreover, some possibility of allergies, namely acute hypersensitivity reactions—such as angioedema, dyspnea and bronchospasm—in sensitized individuals can cause anaphylactic reactions [39]. Considering that no threshold dose was established for allergic reactions, exposure to eliciting allergens, such as proteinaceous compounds, must be avoided as much as possible by reducing their presence through purification steps during the manufacturing process of E120 [39].

#### 2.1.4. Chlorophylls, Chlorophyllins, and Copper Complexes of Chlorophylls and of Chlorophyllins

**Figure 4:**
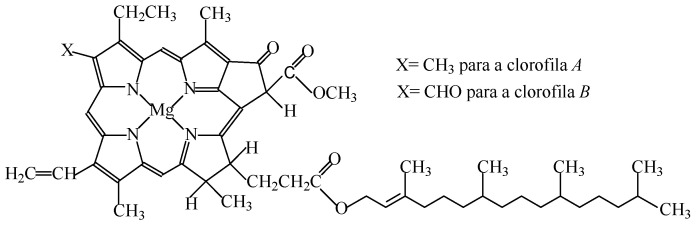
Structural formula of chlorophylls *a* and *b*.

The chloroplasts of higher plants have two types of chlorophyll (*a* and *b*), which are insoluble in water, soluble in alcohol, and sensitive to light, pH, oxygen and heat (Figure 4). Chlorophyll *a* prevails, but degrades more easily. 

Chlorophyll, with the code E140(i), can be extracted with acetone, methyl ethyl ketone, dichloromethane, carbon dioxide, methanol, ethanol, propan-2-ol and hexane [7]. Heating foods containing chlorophyll (i.e., scalding vegetables before freezing or before they are canned) might lead to a colour change (to a more “pale” green), due to the loss of the magnesium atom in the chlorophyll molecule (which therefore determines pheophytins synthesis). Thus, the instability of chlorophylls determines their limited use as food additives, despite the absence of toxicity for the amounts usually consumed [1,2]. 

Chlorophyllins (E140(ii)) are water-soluble, and are the simplest derivatives of lipid-soluble chlorophylls (E140i) that are obtained through partial breaking. Chlorophyllins, obtained by the saponification of the solvent-extracted yield from edible plant material, break the ester–phytol bond, increasing the polarity of the derived products. However, as both colorants are rather unstable, the food industry favors their substitution by the lipid-soluble cupric chlorophyll (E141(i)) and water-soluble chlorophyllin complexes (141(ii)). These derivatives result from the replacement of Mg by Cu atoms, and determine a higher colour stability. The insertion of Cu into the chlorophyll molecule stabilizes the structure, and the green coloration does not change with the processing conditions or storage time of the coloured food.

All of these additives give colours varying among olive green, dark green, blue-green and dark blue to food products (Table 1) [7].

The colourants E140 and E141 can be used in most food products, namely in naturally green fruits to be preserved in syrup, vegetables preserved in vinegar, in oil or in brine (except olives), seasoning, jams and jellies, marmalades, desserts, ice creams, flavoured fermented milk products, confectionery including breath refreshing and chewing gum, pastry and fine bakery products, decorations and coatings, Sage Derby cheese, edible cheese rind, flavoured non-alcoholic drinks, alcoholic beverages, appetizers, fish paste and crustacean paste, precooked crustaceans, and smoked fish [3].

Chlorophylls are natural dietary constituents which are present at relatively high concentrations in a large amount of foods that, upon ingestion, are excreted in faeces. Accordingly, its consumption as a food additive is not of safety concern [42]. Besides this, because the Cu present in cupric chlorophyll (E141(i)) and chlorophylline complexes (141(ii)) is firmly bound, the consumption of these food colours causes no adverse effects to human health. Eventually, as western diets are usually deficient in Cu, the intake of these food colours might even trigger health benefits, but could be problematic for people with pathologies that lead to the accumulation of copper in their body [2].

#### 2.1.5. Caramels

Caramels, with the code E150, are one of the oldest and most-often used colourants in foods and beverages. Caramels are often used in the food industry to impart or intensify the yellow or brown colour, being miscible with water (in liquid or powdered forms), but can also disperse in an oil system (producing pastes or emulsions). Four caramel food colours can be used—E150a, E150b, E150c, and E150d—and are prepared by caramelization (i.e., the controlled heat treatment of carbohydrates with food-grade reactants) but differ in some of the raw materials used in their preparation (Table 2). During caramelization, carbohydrates incompletely decompose, dehydrate and polymerize at high temperatures (which is closely related to the temperature and type of carbohydrates), producing a mixture of chemical substances without specific compositions. For instance, at 160 °C, sucrose forms glucose and fructan, whereas at 185–190 °C isotopecane ((C_12_H_24_O_10_)n) is synthetized, and, at about 200 °C, polymerization develops caramel alkane ((C_24_H_36_O_18_)n) and caramel olefins ((C_36_H_50_O_25_)n). Additionally, caramel alkyne ((C_24_H_36_O_13_)n) occurs at 200 °C or more. All of these food colour caramels are described as dark brown to black liquids or solids, with an odour of burnt sugar [7].

The colourants E150a and E150b can be used in most food products, namely in flavoured drinks, black beer, whiskey, brandy, rum, liqueur wines, flavoured drinks based on wine, cider, vinegar, flavoured fermented milk products, desserts, edible ices, edible cheese rind, preserves of fruits, malt bread, breakfast sausages, soups, fish paste and crustacean paste, sauces and puddings, and confectionery, including breath refreshing and chewing gum [3]. 

The colourants E150c and E150d are used in decorations and coatings, pastries and fine bakery products, flavoured fermented milk products, flavoured drinks, marmalades, jams and jellies, desserts, confectionery including breath refreshing and chewing gum, edible cheese rind, edible ices, sausages, malt bread, salami and pâtés, hamburgers with vegetables, vinegared vegetables, brine or oil, appetizers, fish paste and crustacean paste, precooked crustaceans, cheese rinds and edible casings, and breakfast cereals flavoured with fruits [3].

Considering genotoxicity, carcinogenicity, reproductive and developmental toxicity, an acceptable daily intake of 300 mg/kg_bw_ for E 150a, E 150b and E 150d was indicated. Moreover, for E150c, only 100 mg/kg_bw_ was considered due to immune toxicity implications [43]. 

The caramels E150a and E150b do not appear to have side effects on health, but at high doses the consumption of the additives E150c and E150d might induce cramps, decreased appetite and white blood cells (symptoms found in experiments with rats), and gastrointestinal disorders [16]. Eventually, the main recurring problem regarding the safety of caramels concerns the presence of 4-methylimidazole (an impurity produced upon ammonia processing), which leads to convulsions when fed to rats, mice and chicks [11]. It was further reported [11] that ammoniated caramels can also negatively affect the levels of leukocytes (especially lymphocytes) in animals, which inhibits the absorption of vitamin B_6_ in rabbits.

#### 2.1.6. Vegetable Carbon

Vegetable carbon, also known as “vegetable black” (E153), is insoluble in water and organic solvents [7], and is produced from green bamboo refined from through a high-temperature carbonization process with steam activation. The steam activation is achieved by charring the vegetable fibers of vegetable materials, such as wood, cellulose residues, peat, coconut husks and other fruits. The obtained residues are ground into small particles, with glycerin or glucose added for use in food products. Therefore, this additive, which takes the form of an odourless black powder, essentially consists of finely divided carbon, but can also contain small amounts of nitrogen, oxygen and hydrogen, and can adsorb other substances after preparation.

E153 is rarely utilized alone in foods (as it offers an intense black color), but is suitable for vegetable foods and morbier cheese, fish paste and crustacean paste, precooked crustaceans, smoked fish, flavoured drinks, flavoured fermented milk products, desserts, edible ices, edible cheese rind, and confectionery, including breath refreshing and chewing gum [3].

The available toxicological data is too limited to establish an acceptable daily intake for vegetable carbon, but may be emended to include a restriction of the particle size (lower than 100 nm) to exclude the presence of nanoparticles [44]. Although the genotoxicity and carcinogenicity of carbon blacks of hydrocarbon origin has been related to their content of polycyclic aromatic hydrocarbons, the margins of exposure for benzo[*a*]pyrene exposure from vegetable carbon were considerably higher than those estimated from the dietary benzo[*a*]pyrene exposure [44]. Thus, the use of levels of vegetable carbon (E153) containing less than 1.0 µg/kg residual carcinogenic PAHs, expressed as benzo[*a*]pyrene, is not of safety concern [44]. Besides this, vegetable carbon is an inert substance which is not absorbed from the gastrointestinal tract [44]. 

#### 2.1.7. Carotenes

**Figure 5:**
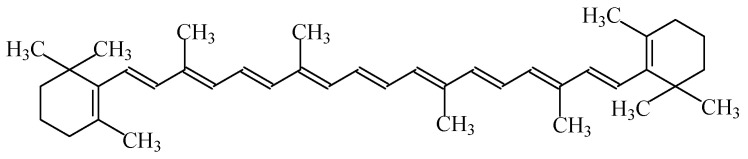
Structural formula of β-carotene (β,β-carotene), the main component of E160a.

Carotenes are hydrocarbons with a skeleton of 40 carbon atoms; like xanthophylls, they belong to the family of carotenoids. Most of the physical and chemical characteristics of β-carotene (Figure 5) are similar to the other family members of carotenoids [1].

Food colour carotenes (E160a), also known as “CI food orange 5”, include four subcategories of carotene food colours (β-carotene—E160a(i), plant carotenes—E160a(ii), β-carotene from *Blakeslea trispora*—E160a(iii), and algal carotenes—E160a(iv)). All of these colourants give an orange colour to food products [7]. β-Carotene E160a (i) is synthetically produced or isolated from natural sources, including krill, in a mixture with other carotenoids (El60a(ii)—natural carotenoid extracts). Besides this, E160a(iii) and E160a(iv) are obtained through fermentation with *Blakeslea trispora* and *Dunaliella salina*, respectively.

They are soluble in nonpolar solvents and fats but insoluble in water. This feature could restrict the use of carotenoids in general, or β-carotene in particular, to the staining of fats (such as margarines). However, when combined with surfactants, they lead to the formation of micro-emulsions which are suitable for staining. These compounds are very sensitive to oxygen and light, but in the absence of these factors, carotenoids are stable in food products even at high temperatures. The degradation of carotenoids is further accelerated by free radicals synthesised during lipid oxidation.

The subcategories integrated in the group of food colours labelled E160a can be used in a large amount of products, namely orange, yellow or whitish coloured cheese, edible cheese rind, unflavoured melted cheese and other processed cheese, butter, fat and oil emulsions and other spreadable fats, sausages, salami and pâtés, preserves of fruits, jams and jellies, marmalades, confectionery including breath refreshing and chewing gum, pastry and fine bakery products, desserts, breakfast cereals flavoured with fruits, flavoured dairy products, edible ices, dried potato granules and flakes, sauces and seasonings, seasonings, fish paste and crustacean paste, precooked crustaceans, soups, smoked fish, and alcoholic beverages [3,9].

No acceptable daily intake has been established for the use of mixed carotenes and β-carotene as food colours [45]. Indeed, if its use does not surpass the amount ingested from regular consumption in foods (considering their natural occurrence, 5–10 mg/day), side health effects are not a concern.

Excess carotene can be stored in the liver and fat of the human body, and can be metabolized to vitamin A. Besides this, β-carotene intake is prescribed for people suffering from increased photosensitivity (erythropoietic protoporphyria). It also helps prevent cognitive decline and—as an antioxidant—eliminates toxins, preventing their accumulation in the body while enhancing the resistance to negative radiation exposure; it is also an effective prophylactic agent against cancer and cardiovascular diseases [16,45]. Nevertheless, the consumption of an excessive amount of E160a supplement for people at risk of cancer (smokers, people who drink alcohol, and workers in the asbestos industry) is not recommend. Accordingly, although there are no studies proving that this applies to the entire population as a whole, considering the level of supplemental intake of β-carotene for which epidemiological studies did not reveal any increased cancer risk, its consumption must remain below 15 mg/day [45]. Besides this, an excess of carotene in the body can lead to the disease of carotenemia, but as this chemical entity has a low toxicity, this is not considered a dangerous pathology, but it can change the skin tone to a more yellow colour [45]. The occurrence of urticaria is also possible due to an allergic reaction to β-carotene [16].

#### 2.1.8. Annatto Bixin and Annatto Norbixin 

Annatto bixin E160(i) and annatto norbixin E160(ii) can be obtained by extraction from the spiney seed pods of *Bixa orellana* L. (also known as “Annatto” or ‘Achiote’ in large parts of South America, and as ‘Urucum’ in Brazil), and provide a yellow-to-red colour to food products [10]. 

Bixin, which is a carotenoid, is extracted from the seed using hot vegetable oil. However, bixin is only soluble in vegetable oil at low percentage rates. Thus, stronger products can be obtained using bixin suspensions, by carrying out repeated extractions of annatto seeds (yielding bixin concentrations of ca. 4% or greater). Oil-soluble bixin is a yellow color, but its suspensions are a deep, vivid red-orange. By saponification, the methyl ester of bixin is cleaved, forming norbixin products, which are water-soluble colourants. Like bixin, norbixin varies in hue from yellow to orange, depending on the usage rate and application.

**Figure 6:**
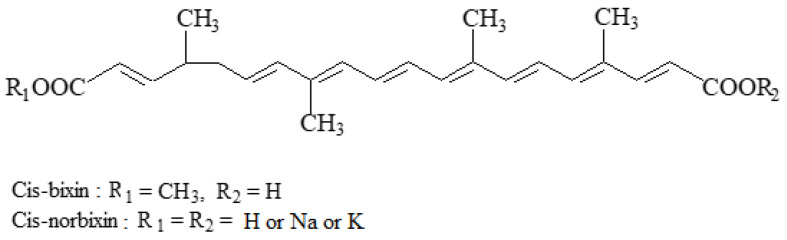
Structural formulae of bixin and norbixin colourant components.

In spite of their similar structures (Figure 6), bixin and norbixin have different physicochemical characteristics [10]. These additives replaced solvent-extracted bixin and norbixin food colours (i.e., the alkali extract of annatto E160b(ii) and oil-extracted annatto E160b(iii), which were previously authorized) [3,7]. 

The food colours annatto bixin E160(i) and annatto norbixin E160(ii) can be used in ripened cheese, flavoured melted cheese, and other processed cheese, flavoured fermented milk products, the decorations and coatings of cakes, desserts, jams and jellies, marmalades, processed potato products, snacks, smoked fish, edible cheese rinds, noodles, batters for coating, some confectionery products, sausages, pâtés, terrines, chorizo, salchichon and other meat products, soups and broths, and sauces [10]. 

E160(i) can also be used in some fat and oil emulsions and other spreadable fats, and flavoured and spirit drinks. 

E160(ii) can further be used in edible ices, breakfast cereals flavoured with fruits, fine bakery, and some alcoholic drinks with less than 15% alcohol [10].

An acceptable daily intake of 6 mg bixin/kg_bw_ and 0.3 mg norbixin/kg_bw_ has been reported [46]. In this context, several studies in humans showed that the intake of norbixin was much lower than bixin (7% and 93%, respectively), but as the major metabolite recovered in plasma is norbixin (more than 90%), the in vivo conversion of bixin to norbixin was suggested [47]. Moreover, it has been estimated that bixin is below the acceptable daily intake for all population groups, whereas for norbixin an exceedance was observed for the extension of use at the 95th percentile for toddlers and children [46]. Besides this, in humans it was considered that norbixin is a metabolite of toxicological relevance following the oral administration of bixin [46].

#### 2.1.9. Paprika Extract

**Figure 7:**
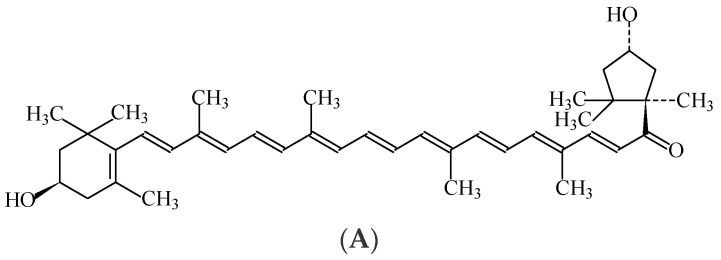

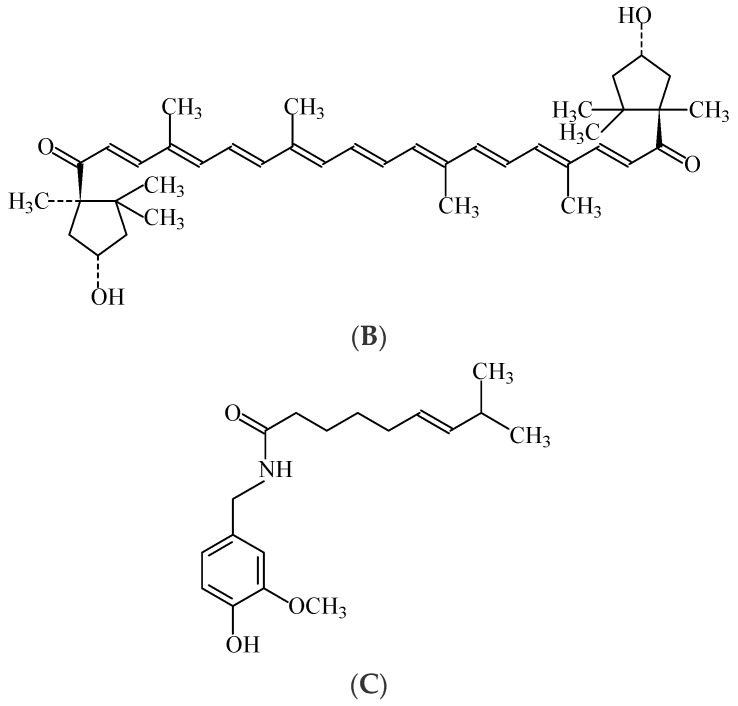
Structure formulae of capsantin (**A**), capsorubin (**B**) (colored components of pepper extract), and capsaicin (**C**), the main flavouring component of chili extract.

Paprika extract, also known as “capsanthin” or “capsorubin”, is a natural food colouring (E160c) in the form of a viscous, dark-red liquid. Capsanthin and capsorubin (Figure 7) are the colourings that impart the yellow-to-orange hue characteristics of paprika. Paprika is extracted with acetone, ethanol, methanol, hexane, dichloromethane, ethyl acetate or carbon dioxide from ground fruits, with or without seeds, from the natural strains of *Capsicum annuum* L. that contain capsanthin (C_40_H_56_O_3_), capsorubin (C_40_H_56_O_4_), and other lesser quantities of coloured compounds, namely xanthophyll, β-carotene and capsaicin [7]. Paprika extract also contains a large amount of capsaicin (C_18_H_27_NO_3_), which is a flavour component. This red spice imparts flavour, and the paprika color compounds can be solvent extracted to synthesize paprika oleoresin, a purified form of the colouring compounds. Paprika and paprika oleoresin are stable to heat but sensitive to light and alkaline conditions, and are insoluble in water.

Paprika extract can be used in most food products, namely jams and jellies, marmalades, desserts, edible ices, confectionery including breath refreshing and chewing gum, cured orange cheese, edible cheese rinds, flavoured melted cheese, seasoning, breakfast cereals flavoured with fruits, sausages, sweets, pâtés, fish paste and crustacean paste, fish eggs, smoked fish, sauces, and precooked crustaceans [3].

The bioavailability of capsanthin and capsorubin from paprika extract is very low, and does not raise genotoxic or carcinogenic concern. Moreover, based on the lack of genotoxic potential, and on the no-observed-adverse-effect level for histopathological changes, an acceptable daily intake of 24 mg/kg_bw_ for paprika extract has been indicated [48]. 

#### 2.1.10. Lycopene

**Figure 8:**
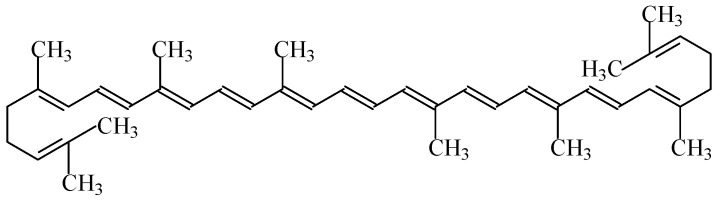
Structural formula of lycopene (E160d).

Lycopene, a bright red carotenoid, is a symmetrical tetraterpene (i.e., assembled from eight isoprene units). In its natural, all-*trans* form, the molecule is long and straight, constrained by its system of 11 conjugated double bonds (Figure 8), which reduces the energy required for electrons to transition to higher energy states, making possible the absorption of visible light with progressively longer wavelengths. Thus, as it absorbs the longest wavelengths of visible light, it displays a red colour. Lycopene (C_40_H_56_), exists as synthetic lycopene (E160d i), as lycopene from red tomatoes (E160d ii), and as lycopene from *Blakeslea trispora* (E160d iii, also called “natural yellow 27”). The food colours E160d-i and E160d-iii are red crystalline powders, whereas E160d-ii is a viscous liquid with a dark red colour. Lycopene is insoluble in water, but freely soluble in chloroform [7]. 

Lycopene, the main colouring product of red tomatoes (which also have small amounts of other carotenoid pigments and oils, fats, waxes and naturally occurring aromas) is obtained by extraction with ethyl acetate, acetone, dichloromethane, carbon dioxide, ethanol, methanol, 2-propanol and hexane, followed by the subsequent removal of the solvent.

This food colour has been used in processed cheese, confectionery including breath refreshing and chewing gum, decorations, the coatings and fillings of cakes and pastries, jams and jellies, marmalades, cookies, desserts, edible ices, flavoured fermented milk products, flavoured drinks, sauces, seasonings, meal substitutes, smoked fish and salmon substitutes, appetizers, edible cheese rinds, batters for coating, fine bakery wares, soups and alcoholic beverages [3].

The intestinal absorption of lycopene requires its combination with bile salts and fat to form micelles. Accordingly, an acceptable daily intake for lycopene of 0.5 mg/kg_bw_ from all sources has been indicated [49]. However, high consumers of foods containing lycopene in certain groups of the population, such as preschoolers and schoolchildren, may exceed that value. Several reports have also indicated that lycopene, as a food colourant, is non-toxic [16,49]. Moreover, other studies have pointed out that intolerance or allergic reactions to dietary lycopene (which may promote diarrhea, nausea, stomach pain or cramps, gas, and loss of appetite), an increased risk of bleeding when taken with anticoagulant drugs, the development of low blood pressure (in case of interaction with drugs), or lycopenemia (i.e., a discoloration of the human skin due to high intakes of lycopene) might occur [50]. Alternatively, some positive health effects at a cardiovascular level (namely on elevated blood lipids and pressure) have been suggested [51,52,53].

#### 2.1.11. β-Apo-8′-carotenal (C 30)

**Figure 9:**
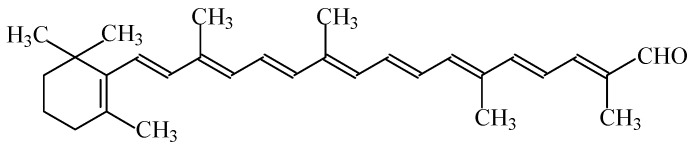
Structural formula of β-apo-8′-carotenal (E160e).

The carotenoid β-apo-8′-carotenal (C 30), also called “Cl 6 food orange” (E160e), is a natural, but chemically modified, food colouring (C_30_H_40_O), ranging from orange to dark red (Figure 9). This carotene aldehyde is resistant to temperature, does not decompose from light, preserves the shelf life of products, and restores products’ colour after heat treatment.

β-apo-8-carotene consists of dark-purple crystals with a metallic luster or a crystalline powder, and can be isolated from spinach, oranges, grass, tangerines or calendula. The diluted or stabilized forms of E160e are solutions or suspensions of β-apo-8′-carotenal (C 30) in edible oils and fats, emulsions and powder products, which are dispersible in water and contain different proportions of cis/trans isomers. 

E160e is used in flavoured drinks, ice cream, desserts, confectionery including breath refreshing and chewing gum, crackers, flavoured fermented milk products, edible cheese rind, edible ices, sauces, seasoning, fish paste and crustacean paste, precooked crustaceans, meal substitutes, appetizers, soups, and alcoholic beverages [3]. 

An acceptable daily intake for β-apo-8′-carotenal (C 30) of 0.05 mg/kg_bw_ was initially indicated [54], but, following additional studies in rats, a new value of 0.3 mg/kg_bw_ was indicated [55]. Nevertheless, no relevant genotoxicity or other side health effects were identified. Ethyl and methyl β-apo-8′-carotenate, free and esterified vitamin A, and vitamin A alcohol were identified as metabolites of β-apo-8′-carotenal (C 30). Nevertheless, dietary β-apo-8′-carotenal (C 30) is extensively metabolized in intestinal cells, in pathways similar to the metabolism of retinol, and is not directly absorbed from the diet [56].

#### 2.1.12. Lutein

**Figure 10:**
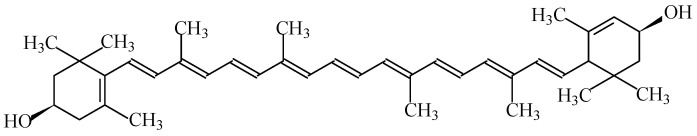
Structural formula of lutein (E161b).

Lutein is a xanthophyll (C_40_H_56_O_2_) of natural origin used as food colouring (E161b), which appears in the form of a dark, yellowish-brown liquid, and gives a reddish-yellow colour to food products. This food colour (Figure 10), which is fat-soluble and a powerful anti-oxidant [57], is obtained by extraction with acetone, methyl ethyl ketone, ethanol, methanol, 2-propanol, hexane, dichloromethane or carbon dioxide [7] from natural varieties of edible fruits and plants, grass, lucerne (alfalfa) and *Targets erecta* (i.e., although lutein also exists in eggs, it is not extracted from this product). Nevertheless, after removal, lutein extracts can also contain fats, oils and waxes from the original plants. 

Lutein has been used in sauces, seasonings, soups, some alcoholic beverages, marmalades, flavoured drinks, jams and jellies, cheese rinds and processed cheese, flavoured dairy products, edible cheese rinds, edible ices, desserts, confectionery including breath refreshing and chewing gum, pastry and fine bakery products, appetizers, sauce, fish paste and crustacean paste, and precooked crustaceans [3].

Considering the absence of reproductive toxicity and chronic toxicity/carcinogenicity, an acceptable daily intake of 1 mg/kg_bw_ has been indicated for lutein [58]. Lutein is consumed daily in natural foods, lowering the risk of cataracts and macular development [59,60]. Alternative medicine claims that lutein supplements also protect against colon and breast cancer, diabetes and heart disease [61,62]. In this context, lutein quenches peroxy radicals and shows antioxidant features against oxidative damage. Besides this, lutein can interact with the mutagens 1-nitropyrene and aflatoxin B1 [63], and can display a protective impact on preneoplastic colorectal adenocarcinoma lesions [64]. As a food additive, there are no reports of side health effects for E161b.

#### 2.1.13. Canthaxanthin

Canthaxanthin (C_40_H_52_O_2_) is a keto-carotenoid pigment which is widely distributed in nature (namely, in flamingo feathers, koi carp skin and crustacean shells) that was firstly isolated in edible mushrooms. This food colour (E161g), also named “Cl food orange 8” and known as β-Carotene-4,4′-dione and 4,4′-dioxo-β-carotene [7], is a potent lipid-soluble antioxidant [65,66]. Although it is a natural product, for food colour purposes, canthaxanthin is usually synthesized. 

**Figure 11:**
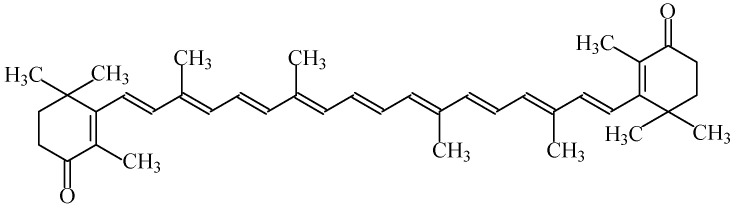
Canthaxanthin’s structural formula (E161g).

This food colour (E161g) takes the form of deep-violet crystals, or crystalline powder, and is sensitive to oxygen and light; therefore, it must be kept in a light-resistant container under inert gas. It is also insoluble in water and ethanol, practically insoluble in vegetable oils, and slightly soluble in acetone [67]. Usually, E161g (Figure 11) is applied in the form of suspensions, solutions or powders which are dispersible in water, containing different ratios of cis/trans isomers. 

E161g can only be used in Strasbourg sausages at a concentration of 15 mg/kg [68], but is widely used in poultry (broiler, laying hens) as a feed additive [69].

Several side health effects have been described, namely allergic reactions in the form of urticaria and Quinche’s edema, as well as the accumulation of crystalline deposits in the retina [16,69]. Nevertheless, accumulation in the retina was only found in a limited number of people that consumed very high amounts of canthaxanthin via sun-tanning pills (i.e., after stopping the pills, the deposits disappeared, and health was fully recovered). Therefore, this crystal synthesis in the retina is not associated with detectable functional changes and temporary or permanent visual loss [70]. Moreover, although some potential benefits on consumers’ health have been shown through in vitro studies (which require validation with in vivo models), canthaxanthin created through the enrichment of LDL might protect cholesterol from oxidation. Its free radical scavenging and antioxidant properties might also trigger the induction of catalase and superoxide dismutase, and its canthaxanthin’s immunomodulatory activity can enhance the proliferation and function of immune cells [1,2]. In this context, an acceptable daily intake for canthasantin of 3 mg/kg_bw_ was indicated [69]. Within this range, the consumption of canthaxanthin that is derived from egg yolks, poultry and fish does not normally exceed the acceptable daily intake (i.e., considering that the maximum residue levels of this molecule do not surpass 30 mg/kg for egg yolk, 15 mg/kg for poultry liver, 2.5 mg/kg for poultry skin/fat, 10 mg/kg for salmon and 5 mg/kg trout flesh) [71,72,73].

#### 2.1.14. Beetroot-Red

Beetroot-red, also named “betanin” or “beet-red”, is a natural food colour (E162) displaying red to dark-red colours. It is a red glycosidic food dye obtained from the roots of red beets (*Beta vulgaris* L. var. rubra), by pressing crushed beets, or by the aqueous extraction of shredded beet roots (the concentration of which can reach about 300–600 mg/kg) and subsequent enrichment in the active principle [7].

**Figure 12:**
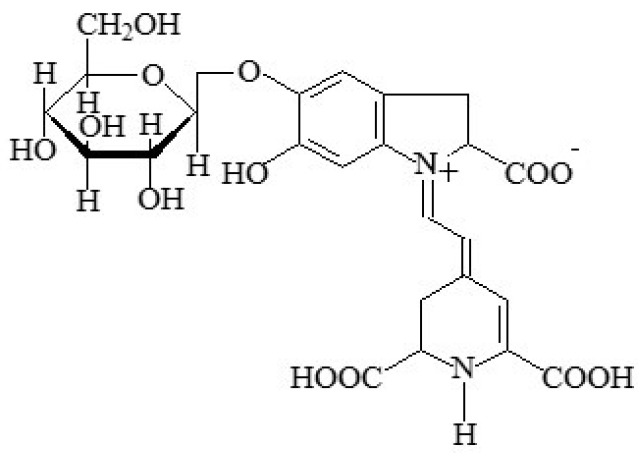
Structural formula of betanin, the main coloured component of beet red (E162).

The colour principle consists of several pigments belonging to the betalain class. Betacyanins (red), with betanin (Figure 12) being the main component (75–95%), are the main coloured components. Betaxanthin (yellow) and some degradation products (light brown) may also occur in smaller amounts. In addition to the pigments, some naturally occurring sugars and proteins can also occur in the beet juice, but its concentration and refinement can remove most of them. 

**Figure 13:**
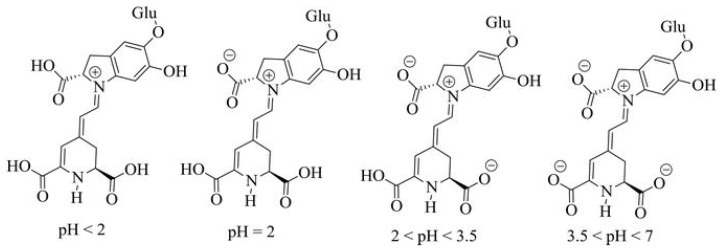
Structures of betanin as the pH varies [74].

Betanin is a red or dark red (depending on pH—between four and five it is bright bluish-red, becoming blue-violet as the pH increases, and at alkaline levels its degradation by hydrolysis produces a yellow-brown color—Figure 13) liquid, paste, powder or solid substance, which is soluble in water and insoluble in ethanol [74].

This food colour is especially used in food products for children, such as ice cream and flavoured dairy products. Additionally, it is applied in a large number of other food products, namely sausages, salami and pâtés, marmalades, jams and jellies, vegetables in vinegar, brine or oil, breakfast cereals with fruits, confectionery including breath refreshing and chewing gum, pastries and bakery products, soups, seasonings, corn, flavoured melted cheese, edible cheese rind, fish paste and crustacean paste, precooked crustaceans, flavoured drinks, desserts, and alcoholic drinks [3]. In general, besides acidic conditions, E162 also degrades when submitted to light, heat and oxygen, and is thus mostly used in frozen products, products with a short shelf life, or solid products in a dry state. However, it can be submitted to pasteurization if the food product has a high content of sugars, and in spite of its high sensitivity to oxygen in watered products and/or products containing metal ions, in conjunction with ascorbic acid and sequestrants, the degradation can be retarded. Moreover, in dry products, betanin is stable in the presence of oxygen [75].

The acceptable daily intake has not been established [76]. This food colour is poorly absorbed in the intestine, being mostly eliminated in the urine. Although some allergic reactions might develop, no other harmful effect has been reported. Moreover, it has been stated that beetroot is an antioxidant, an antidepressant, antimicrobial, antifungal, anti-inflammatory and diuretic [76,77]. It is also an expectorant and carminative, hepatoprotective, a protector of cardiovascular health, and an inhibitor of lipid peroxidation, while having chemo-preventative effects [77].

#### 2.1.15. Anthocyanins

**Figure 14:**
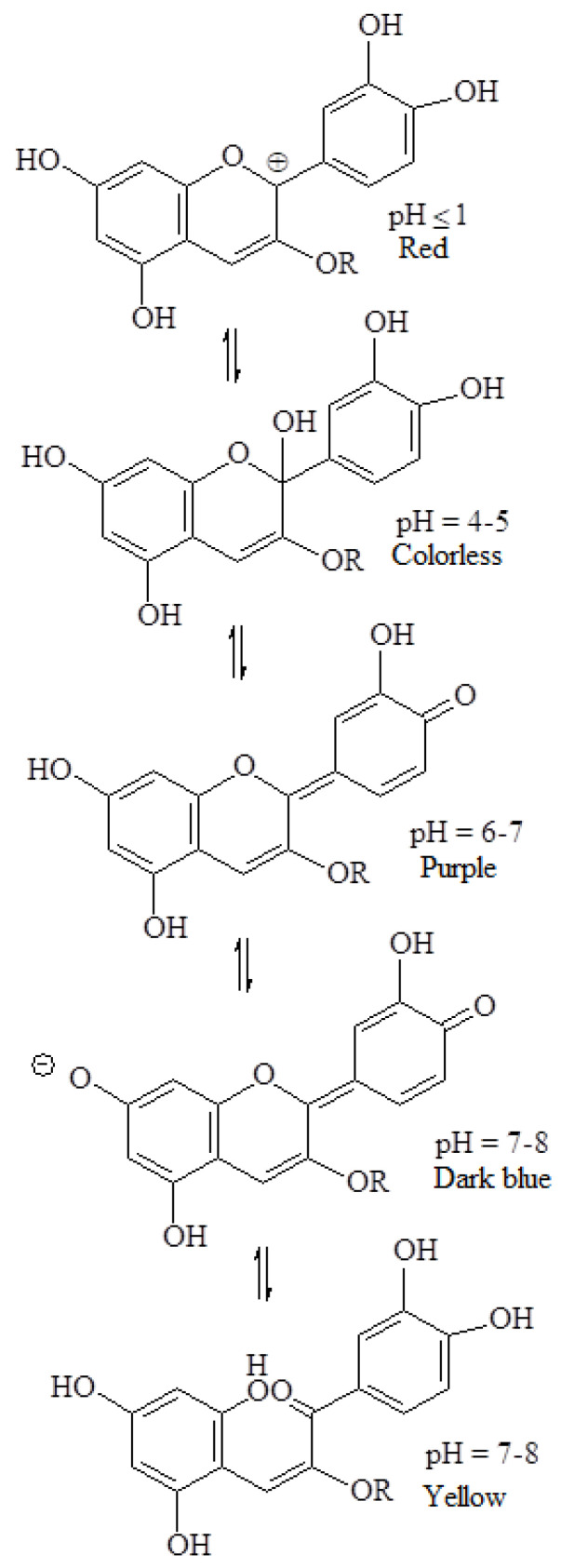
Example of anthocyanin changes due to pH variation [78].

Anthocyanins are a group of natural water-soluble food colours (E163) that take on colours ranging from purple to blue, but can display other colours depending on the pH (Figure 14) [78]. Anthocyanins belong to a parent class of flavonoids synthesized via the phenylpropanoid pathway; although they occur in all tissues of higher plants, they can be obtained from edible vegetables and fruits, such as blueberries, blackberries, strawberries, currants, raspberries, and grapes.

Absorption spectroscopic studies with standardized solutions of cyanidin-3-ramnoglucoside allowed the identification of the pH-dependent equilibria that are responsible for great colour variations. The β-ring hydroxylation status and pH have been shown to mediate the degradation of anthocyanins to their phenolic acid and aldehyde constituents [79]. Additionally, anthocyanins are relatively unstable substances that, beyond having an acceptable behaviour only in an acidic environment, are also affected by temperature, light, oxygen, metal ions, intramolecular association, and intermolecular association with other compounds (namely co-pigments, sugars, proteins, sulphites and degradation products) that can further change their color and stability [80]. 

The most important anthocyanin source in the food industry is the by-products of red wine. Among the anthocyanins extracted can be highlighted cyanidin (C_15_H_11_O_6_Cl), peonidin (C_16_H_13_O_6_Cl), malvidin (C_17_H_15_O_6_Cl), delphinidin (C_15_H_11_O_7_Cl) and petunidin (C_16_H_13_O_7_Cl) [7]. In this context, it must be pointed out that petunidin is resistant to degradation at pH 8, and may be effectively used as a food colourant [81]. In addition to anthocyanins, the extracts may contain sugars, organic acids (such as tartaric acid in grapes), minerals, and other naturally occurring substances in the fruits or vegetables from which they are extracted.

E163 can be used in most food products, namely flavoured dairy products, ice creams, desserts, marmalades, jams and jellies, breakfast cereals flavoured with fruits, cheese products, confectionery including breath refreshing and chewing gum, fish paste and crustacean paste, precooked crustaceans, preserves of vegetables, canned fish, canned red fruits, flavoured drinks, and liqueurs and other alcoholic beverages [3].

The acceptable daily intake of anthocyanins has not been established [82]. When ingested, it is partially destroyed by the intestinal flora, or eliminated by urine (in a small part) or by bile, after some transformations [83,84]. However, eventually metabolites of ingested anthocyanins are reabsorbed in the gastrointestinal tract, thereby entering the blood for systemic distribution, and triggering some effects as smaller molecules. Although anthocyanins have in vitro antioxidant properties [85], after their consumption in foods by humans, there is no evidence of antioxidant effects, namely the protection of DNA and lipids from oxidative damage, or any anti-cancer or anti-aging properties [82,86]. Moreover, Voss [16] pointed out that anthocyanins might trigger a few allergic reactions.

#### 2.1.16. Calcium Carbonate

Calcium carbonate (CaCO_3_), also referred as “CI pigment white 18” or “chalk”, is a stable food colouring (E170) that does not require any specific processing to preserve its colouring properties. Additionally, calcium carbonate is also used as an acidity regulator, an anticaking agent (i.e., prevents food particles from sticking together) and a stabilizer (thus maintaining the uniform dispersal of substances in a food). This inorganic salt is a white crystalline or amorphous, odourless and tasteless powder, which is practically insoluble in water and ethanol [7]. Calcium carbonate is a natural mineral derived from earth’s limestone, marble, or the sedimentation of crushed marine shells. For the food industry, the additive E170 is obtained by processing and cleaning chalk deposits.

E170 can be used in most food products, namely in confectionery products including dragees and chewing gum, decorations and coverings, desserts, cocoa and chocolate products [87], ripened cheese, grated or sliced cheese, processed eggs, salt and salt substitutes, seasoning and condiments, fish paste and crustacean paste, fruit juices and fruit nectars [88], similar vegetable products, flavoured fermented milk products, edible ices, and baby foods [3].

The acceptable daily intake has not been specified (thus, *quantum satis* is allowed), but some estimates indicate that exposures to calcium from all sources, including the use of calcium carbonate as a food additive, taken together with intakes of calcium from supplements and from fortified food should be below 2500 mg/day [89]. Thus, considering the trace levels of calcium carbonate used as food additives, there is no evidence of toxicity or carcinogenic effects [89]. In fact, calcium carbonate has a relevant physiological role in the human body, participating in blood coagulation processes, ensuring constant osmotic blood pressure and regulating various intracellular processes. Moreover, excess calcium may cause lactic-alkaline syndrome, which has a highly toxic effect and, in severe cases, can lead to death. A small overdose of calcium carbonate can also trigger hypercalcemia complications that include vomiting, abdominal pain, and changes in mental status.

#### 2.1.17. Titanium Dioxide

Titanium dioxide (TiO_2_), also named “CI pigment white 6”, is a metal oxide extracted from ilmenite, rutile and anatase. Titanium dioxide is use as a white food colouring (E171) due to its brightness and very high refractive index. Like all food colourants, its technological function is to make food more visually appealing, to give colour to food that would otherwise be colourless, or to restore the original appearance of a foodstuff. Titanium dioxide is also a nontoxic antimicrobial (i.e., it has potential bactericidal and fungicidal applications). E171, which is presented in the form of an odourless and tasteless white amorphous powder with pH 7, consists of pure titanium oxide; however, it can be coated with small amounts of alumina or silica to improve its technological properties. E171 is insoluble in water and organic solvents [7].

This additive can be used in most food products, namely in confectionery including breath refreshing and chewing gum, decorations and coatings, flavoured fermented milk products, edible ices, desserts, edible cheese rind, seasoning, fish paste and crustacean paste, and precooked crustaceans [3].

Some carcinogenic effects have been attributed to E171 [16]. According to Jovanovic [90], TiO_2_ can be absorbed by the gastrointestinal tract, and can bioconcentrate, bioaccumulate, and biomagnify in the tissues of mammals and other vertebrates. Besides this, a recent safety assessment of E171 further concluded that, based on all of the available evidence, a definitive concern for genotoxicity could not be ruled out, and given the many uncertainties, this colourant can no longer be consider safe when used as a food additive [91]. Indeed, although the gastrointestinal absorption of TiO2 particles is low, they may accumulate in the body, and potential immunotoxicity and inflammation with E 171 and potential neurotoxicity with TiO_2_, together with the potential induction of aberrant crypt foci in the colon with E 171, may indicate adverse effects [91]. Besides this, TiO_2_ particles have the potential to induce DNA strand breaks and chromosomal damage, but not gene mutations [91]. Nevertheless, in spite of the health risk, the use of this food colouring has not been banned, but an acceptable daily intake has not been established.

#### 2.1.18. Iron Oxides and Iron Hydroxides

Iron oxides and hydroxides (E172) can naturally be found in rusts, or can be artificially produced from iron sulphate. These colourants (Table 3) can be applied to food products, appear in the form of black, brown, red or yellow powders, and are insoluble in water and organic solvents [7].

E173 can be used in most food products, namely in confectionery including breath refreshing and chewing gum, desserts, decorations and coatings, flavoured fermented milk products, edible ices, edible cheese rinds, seasoning, fish paste and crustacean paste [3].

An acceptable daily intake up to 0.5 mg/kg_bw_ has been reported [92]. With regard to the possible side effects of both food colours, the available data indicate that the absorption of iron from oxides is low, and that oxides and hydroxides are of low toxicity in rats and mice. Nevertheless, gastrotoxicity, hepatotoxicity and alterations in gut microbiota have been reported, and most evidence shows oxidative stress as the main mechanism of toxicity [93]. However, the data are not sufficient to draw conclusions regarding E172’s safety in humans, and additional studies are needed [92].

#### 2.1.19. Aluminum

Aluminum, also referred as “CI pigment meta”, can be used as a food colouring (E173l), being a silvery-grey powder or tiny sheets which are insoluble in water and in organic solvents [7]. Aluminum powder consists of finely divided particles that eventually can be carried out in the presence of edible vegetable oils or fatty acids, and are used as food colours. This dye, which has a very limited use, can be applied in the external coating of sugar-based confectionery products for the decoration of cakes and pastries [3].

Regarding side effects, according to Voss [16], in high doses it is toxic to nerve cells (i.e., it triggers Alzheimer’s disease) and mineral metabolism. Besides this, is not recommend for people with kidney problems. 

#### 2.1.20. Silver

Silver food colour (E174) is present in its elemental form, and is chemically obtained from the electrolysis of silver ore. It is a soft, white and lustrous transition metal, which exhibits the highest electrical conductivity, thermal conductivity, and reflectivity of any metal. This coloured powder, or straw, is also a bactericide and a water-insoluble substance, and is only of use in coatings and decorations for cakes and sweets, liqueurs, and drinking water disinfection [3]. Its acceptable daily intake has not been specified, but at a physiological level the elimination of E174 through the kidneys is slow, which can induce side effects of deposits at the tissue level [16]. For instance, a high intake (a few grams) leads to poisoning. However, there is a lack of data regarding the toxicity and carcinogenic potential of the use of silver as a food additive [93].

#### 2.1.21. Gold

Gold can be applied as a food colour (E175), giving rise to a very unreactive metallic surface colour. It occurs in the form of golden-coloured powders, or straws, and is used in liqueurs and in the outer coating of confectionery and chocolate decorations [3]. The acceptable daily intake for E175 has not been specified, as there are very limited data on the absorption, distribution, metabolism and excretion of elemental gold (as well as the toxicological aspects linked to its use as a food colour). Nevertheless, as elemental gold has a very low solubility, the systemic availability and effects might be low. At a physiological level, some side effects on health—namely disorders of the blood formula—are described [16]. Nevertheless, although there are some studies with rats, the available data did not allow an evaluation of the genotoxic hazard associated to the use of gold as a food colour [94].

### 2.2. Synthetic Food Colours

Synthetic food colours have been increasingly used rather than natural food colours by food manufacturers, as they have several economically relevant traits, such as their low cost; resistance to light, oxygen, and pH changes; and high colour stability. In contrast to natural food colours, which are usually extracted from several natural sources and purified, synthetic food colours are produce by full chemical synthesis or the modification of several precursor compounds. Besides this, they can be used without further transformation, and do not degrade during food processing.

#### 2.2.1. Tartrazine

**Figure 15:**
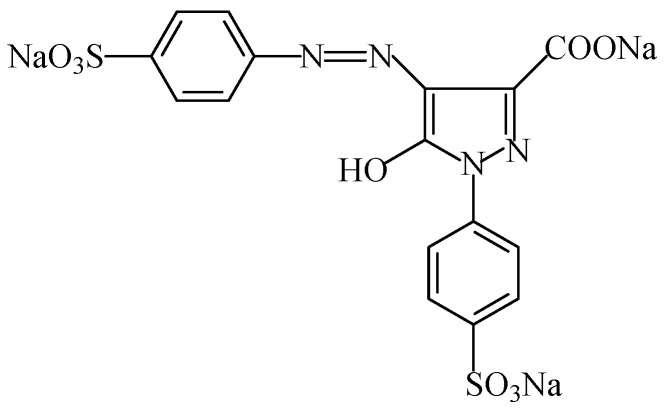
Structural formula of tartrazine (E102).

Tartrazine is a synthetic lemon-yellow azo dye used as a food colour (E102), which is also known as “Cl food yellow 4” [7]. Azo food colours are synthetic, and are prepared from aromatic amines, which contain an azo group of two nitrogen atoms linked together (-N = N-) and linked to aromatic rings [95]. The azo food colours usually have a yellow, red or brown colour. In this context, tartrazine (C_16_H_9_N_4_Na_3_O_9_S_2_), with the chemical name 5-hydroxy-1-(4-sulfonatophenyl)-4-(4-sulfonatophenylazo)-H-pyrazol-3-carboxylate trisodium, usually takes the form of granules or light orange-coloured powders, being soluble in water and sparingly soluble in ethanol [96]. This additive is usually described in the form of the sodium salt (Figure 15), but potassium and calcium salts are also authorized [3,16]. This colourant gives a lemon-yellow colour, but is also use together with blue dyes to bring about a green colour. 

Tartrazine is used as a food colouring in most food products, namely flavoured drinks, alcoholic beverages, seasonings, flavoured fermented milk products, desserts, edible ices, flavoured processed cheese, edible cheese rinds, appetizers, fish roe, fish paste and crustacean paste, precooked crustaceans, smoked fish, pastry and fine bakery products, confectionery including breath refreshing and chewing gum, soft drinks, fruit preserves, processed mushy and garden peas (canned), and other foodstuffs [3,16].

The average daily intake of tartrazine was set as 7.5 mg/kg_bw_ [23]. Tartrazine seems to induce the most allergic and intolerance reactions among all of the azo dyes (particularly among asthmatics and those with an aspirin intolerance); it is further suspected of childhood hyperactivity, and is has been mentioned that it may contain residues of carcinogens [11,16,18]. However, studies to determine the eventual carcinogenic effects of tartrazine on rats showed no carcinogenic changes in the gastric area [97]. Ai-Mashhedy and Fijer [95], working with mice to determine the acute toxicity of tartrazine, also concluded that this food additive is practically nontoxic. Moreover, McCann et al. [14] concluded that exposure to a mixture including tartrazine increased hyperactivity in 3 and 8 to 9 year-old children. Additionally, tartrazine is also suspected of triggering urticaria [98] and angioedema (the swelling of the lips, tongue, throat, and neck caused by the release of histamine in an allergic reaction) [99]. It further acts as a neurotoxin, at least in rats (namely inducing problems with spatial memory) [100].

#### 2.2.2. Quinoline Yellow

**Figure 16:**
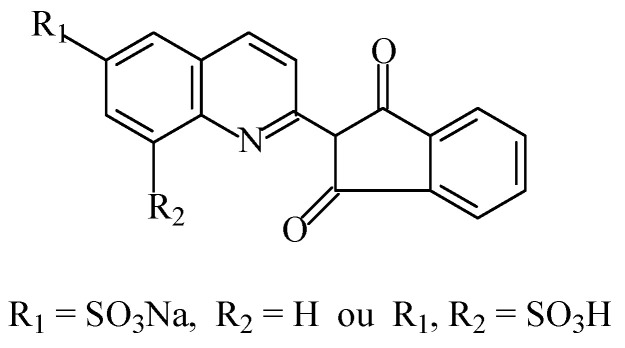
Structural formulae of the main components of quinoline yellow (E104).

The food colouring quinoline yellow (E104), also known as “Cl food yellow 13” [7], belongs to the class of quinophthalone dyes. This colourant consist of a mixture of colours of synthetic origin derived from the quinolone “yellow SS”. Quinoline yellow (Figure 16) is a yellow powder or granule, owing to the presence of sulfonate groups, which is soluble in water and sparingly soluble in ethanol [101]. It is a mixture mostly of disulfonates, but it also has monosulfonates and trisulfonates of 2-(2-quinolyl)indan-1,3-dione. This additive is usually a sodium salt, but potassium and calcium salts are also permitted.

Quinoline yellow has been used in marmalades, jams and jellies, decorations and coatings, pastry and fine bakery products, confectionery including breath refreshing and chewing gum, flavoured fermented milk products, edible cheese rind, flavoured drinks, seasoning, desserts, ice cream, smoked fish, fish roe, fish paste and crustacean paste, shells, cheeses, edible casings, and some alcoholic beverages [3].

This food colouring has not been associated with any significant long-term toxicity, is not genotoxic or carcinogenic, and there is no evidence of adverse effects on reproduction or development [102].

#### 2.2.3. Sunset Yellow FCF

**Figure 17:**
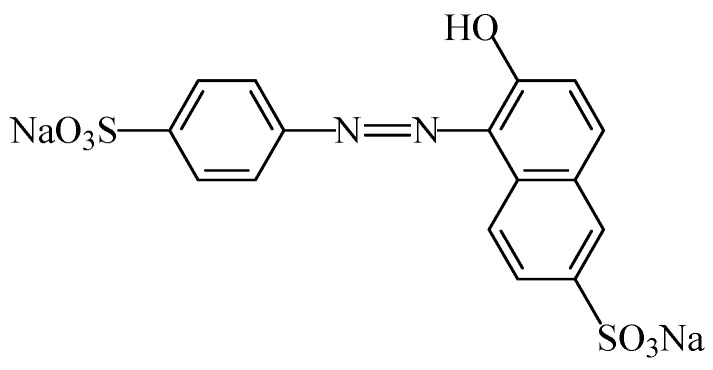
Structural formula of the sunset yellow FCF food colouring (E110).

The food colouring sunset yellow FCF (E110), also known as “orange-yellow *S”* or “Cl food yellow Cl 3” [7], is a petroleum-derived orange azo dye (C_16_H_10_N_2_Na_2_O_7_S_2_) that gives a yellow-orange colour to food products (Figure 17). E110 is an orange-red powder or granule, which is soluble in water and sparingly soluble in ethanol [103]. 

Like other azo food colours, yellow-orange S is usually present as a sodium salt, but potassium and calcium salts are also allowed.

Food colouring E110 comes in the form of orange-to red-granules, or powders, and can be used in a large number of foodstuffs, including flavoured drinks, flavoured fermented milk products, edible ices, edible cheese rind, seasoning, confectionery including breath refreshing and chewing gum, marmalades, jams and jellies, desserts, processed cheeses, flavoured cheeses, cheese rinds, soups, fish roe, fish paste and crustacean paste, precooked crustaceans, smoked fish, and alcoholic beverages [3].

The average daily intake of sunset yellow FCF (E110) is 4 mg/kg_bw_ [102,104]. The use of this colourant can trigger health problems, namely allergic reactions, especially in people with an intolerance to acetylsalicylic acid. It is further suspected of hyperkinesia, and may have residues of potentially carcinogenic substances [16,105]. Studies by Mathur et al. [12,13] further reported significant effects in the lipids of rats exposed for 90 days to 250–1500 mg of Sunset Yellow FCF. Inetianbor et al. [18] also reported the possible occurrence of allergies and asthma, DNA damage and increases tumours in animals, and growth retardation and severe weight loss in animals. Moreover, immunological studies using sunset yellow as a food colouring agent in albino rats showed that humoral immunity is not altered [33], but Yadav et al. [106] reported that sunset FCF alters the functional responses of splenocytes at non-cytotoxic doses.

#### 2.2.4. Azorubine 

**Figure 18:**
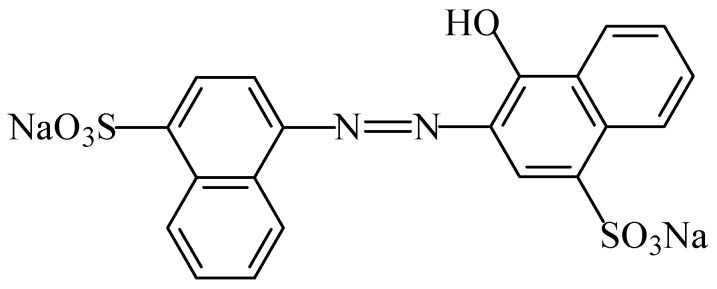
Structural formula of the azorubine food colour (E122).

The food colouring azorubine (E122), also named “carmoisine” and “Cl food red 3” [7], is an azo dye consisting of two naphthalene subunits (C_20_H_12_N_2_Na_2_O_7_S_2_). This synthetic food colour gives to foodstuffs a red colour. This colorant (Figure 18) is soluble in water [107]), slightly soluble in ethanol solution, but insoluble in vegetable oil [108]. Due to the general stability of azo dyes, azorubine is pH and heat stable, and it does not fade away when exposed to light and oxygen [109]. It is mainly used in foods that are heat-treated after fermentation, occurs as red to maroon powder or granules, and is soluble in water and sparingly soluble in ethanol [110]. 

Azurobine has been used in a large number of foodstuffs, namely flavoured drinks, fruit syrups, canned red fruits, ice creams, flavoured fermented milk products, edible ices, desserts, pastry and fine bakery products, confectionery including breath refreshing and chewing gum, soups, sauces, seasoning, seafood, fish roe, fish paste and crustacean paste, precooked crustaceans, appetizers and alcoholic beverages [3].

An acceptable daily intake of 4 mg/kg_bw_ was establish for azorubine [111]. There is no evidence of mutagenic, carcinogenic or teratogenic properties for azorubine with histopathological effects, but in rare situations it seems to cause skin and respiratory allergic reactions [112]. Inetianbor et al. [18] further reported, as possible negative effects of azurobine, DNA damage and tumours in animals. Moreover, Ai-Mashhedy and Fijer [95], working with mice to determine the acute toxicity of carmoisine, only reported a slight toxicity using the Hodge and Sterner scale. 

#### 2.2.5. Amaranth

**Figure 19:**
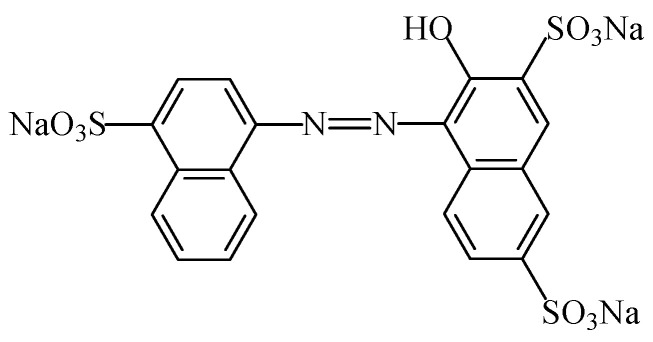
Structure formula of the amaranth colourant (E123).

The amaranth food colouring (E123), also known as “Cl food red 9” (Figure 19), is a modified red azo dye obtained synthetically (C_20_H_11_N_2_Na_3_O_10_S_3_) [7]. This anionic dye is a reddish-brown powder or granule (soluble in water and sparingly soluble in ethanol) that decomposes at 120 °C without melting [113]. Amaranth is used in the form of a trisodium salt, but potassium and calcium salts can also be applied.

The use of amaranth is very restricted, being limited to soft drinks and some alcoholic drinks, including spirits, aperitif wines, and fish roe [3]. An acceptable daily intake of 0.15 mg/kg_bw_ was established for amaranth [114], as this dye can present health problems, namely allergic reactions, asthma (as it is an histamine liberator), or hives [18]. It is also suspected of hyperkinesia, may have residues of potentially carcinogenic substances, and can induce the appearance of calcareous deposits in the kidneys [16]. Immunological studies using amaranth as a food colouring agent in albino rats reported that no alteration of humoral immunity was observed [33]. Amaranth was also associated with cancer, birth defects, stillbirths, sterility, and early foetal deaths when fed to laboratory rats [11].

#### 2.2.6. Ponceau 4R 

**Figure 20:**
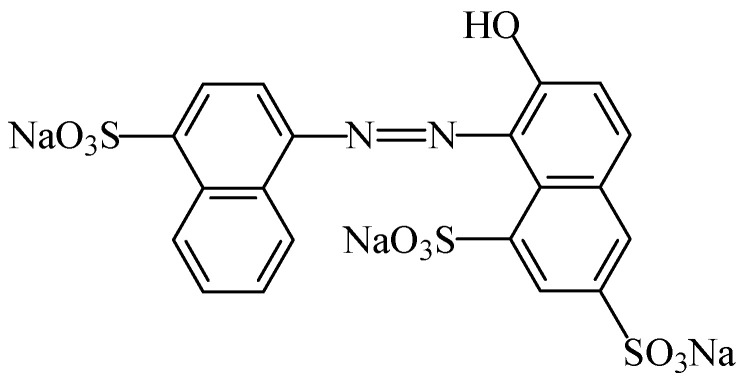
Structural formula of the ponceau 4R colourant (E124).

Ponceau 4R—also known as “cochineal red A”, “Cl food red 7” or “new coccine”—is a synthetic azo food colouring (E124) that gives a red colour to foodstuffs. This food colour (1-(4-sulfo-1-napthylazo)-2-napthol-6,8-disulfonic acid, trisodium salt) is a reddish powder or granule which is soluble in water and sparingly soluble in ethanol [115]. This colourant is manufactured by coupling diazotized naphthionic acid to G acid (2-naphthol-6,8-disulfonic acid), followed by the conversion of the coupling product to the trisodium salt. Although it is usually described as a sodium salt (C_20_H_14_N_2_Na_3_O_10_S_3_) (Figure 20), the use of potassium or calcium salts is also permitted [7]. 

The ponceau 4R colouring is used in a large number of foods to intensify the red colour, such as some sausages, flavoured drinks, flavoured fermented milk products, edible ices, desserts, edible cheese rind, fruit syrups, red fruit preserves, jellies, jams and marmalades, pastries and fine baked goods, confectionery including breath refreshing and chewing gum, soups, appetizers, seasonings, sauces, seafood, fish roe, fish paste and crustacean paste, precooked crustaceans, smoked fish, and alcoholic beverages [3].

The acceptable daily intake for ponceau 4R is 0.7 mg/kg_bw_ [102]. There is no evidence of carcinogenicity, genotoxicity, neurotoxicity, or reproductive and developmental toxicity at the allowed dietary exposures, but surpassing this limit—as with other azo food colours—is known to induce allergic reactions, especially when paralleling intolerance to acetylsalicylic acid, which is suspected of hyperkinesia [16]. In this context, it seems that it may further contain residues of potentially carcinogenic substances, namely unsulfonated aromatic amines [16]. Inetianbor et al. [18] further reported the occurrence of DNA damage, tumours in animals, and effects in asthmatics.

#### 2.2.7. Erythrosine

**Figure 21:**
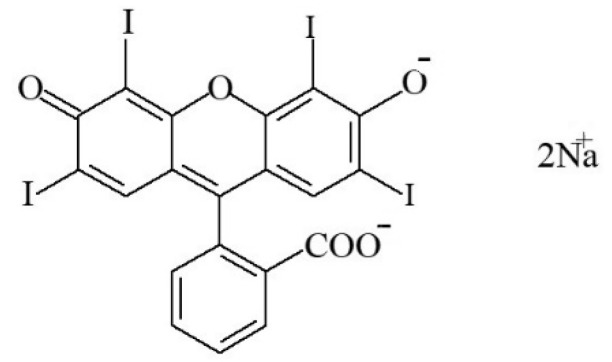
Erythrosine dye’s structural formula (E127).

Erythrosine, also referred to as “Cl food red 14” (C_20_H_6_I_4_Na_2_O_5_·H_2_O), is a xanthene-dye (thus, it is not an azo substance); it is a red food colouring (E127) of synthetic origin [7]. E127 (i.e., a disodium salt of 2,4,5,7-tetraiodofluorescein—Figure 21) is a red powder or granule which is soluble in water and slightly soluble in ethanol [116]. 

This food colour has a very limited use, being applied only in cocktail cherries, candied and bigareaux cherries, syrup and cocktails [3]. The sodium salt of erythrosine is the substance commonly used in the food industry, but potassium and calcium salts are also authorized. 

An acceptable daily intake of 0.1 mg/kg_bw_ was established for erythrosine [117]. The use of this food colouring, which contains high levels of bound iodine (i.e., it is an organoiodine compound, specifically derived from fluorine), has been related with allergic reactions [16] and cancer [18]. Studies with rats and humans showed that only a small portion of erythrosine is absorbed, and that it is excreted almost completely via faeces [117]. Although several in vitro studies showed positive results for genotoxicity, there are negative in vivo genotoxicity studies [117].

#### 2.2.8. Allura Red AC

**Figure 22:**
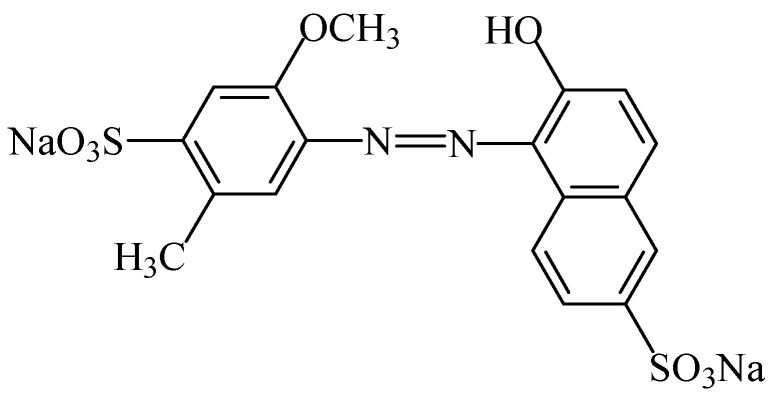
Structural formula of allura red AC (E129).

Allura red AC, also known as “CL Food Red 17”, is a synthetic red azo food colouring (E129) with the chemical formula C_18_H_14_N_2_Na_2_O_8_S_2_ [7]. Although it is usually supplied as a sodium salt, it can also be used in the form of calcium and potassium salts. This food colour (Figure 22), which takes the form of a dark-red powder or granule, is soluble in water but insoluble in ethanol [118]. 

This additive is used in decorations and coatings for pastry products, confectionery including breath refreshing and chewing gum, pastry and fine bakery products, flavoured fermented milk products, edible cheese rinds, desserts, preserves of fruits, ice cream, flavoured drinks, baked crustaceans, seafood, breakfast sausages, appetizers, sauces, seasonings, soups, luncheon meat, replacements, and alcoholic beverages [3].

An acceptable daily intake of 7 mg/kg_bw_ has been indicated [119]. Regarding possible side effects on health, it has been reported that E129 is an allergizing substance which worsens or induces asthma, rhinitis, or hives [18]. There is also the possibility of the presence of carcinogenic residues in this food colouring, and it is suspected of hyperkinesia [16]. McCann et al. [14] further concluded that exposure to a mixture of artificial food colourings, including allura red AC, resulted in increased hyperactivity in 8–9-year old children.

#### 2.2.9. Patent Blue V

**Figure 23:**
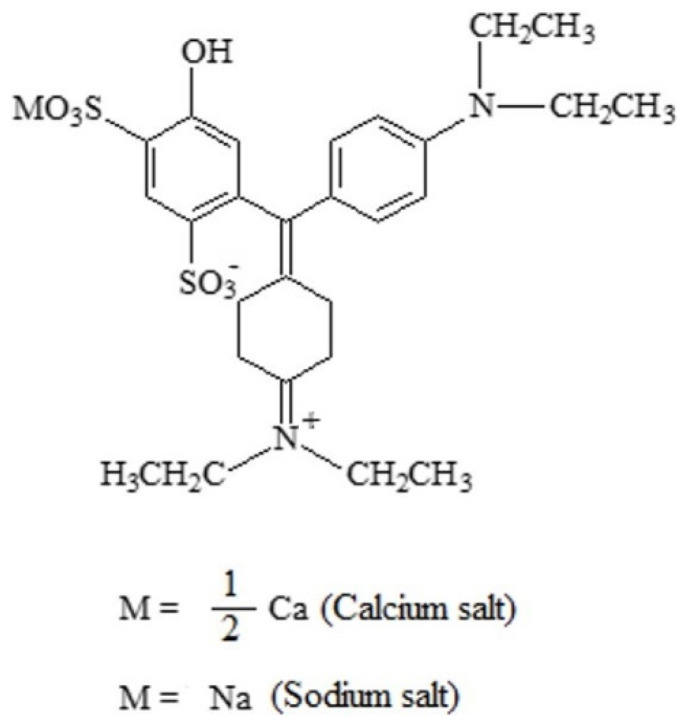
Structural formula of the main components of patent blue V (E131).

The azo food colouring patent blue V (E131), also known as “Cl food blue 5” (Figure 23), is a sky-blue synthetic triphenylmethane dye ([4-(α-(4-diethylaminophenyl)-5-hydroxy-2,4-disulfophenylmethylidene)-2,5-cyclohexadien-1-ylidene] diethylammonium hydroxide), that consists of sodium C_27_H_31_N_2_O_2_S_2_Na or calcium C_27_H_31_N_2_O_2_S_2_Ca_1/2_ salts [7]. The use of the potassium salt is also authorized.

E131 food colour usually takes the form of a blue powder or granule, and is soluble in water and slightly soluble in ethanol [120]. In an aqueous solution, this food colouring is pH-dependent, with its colour varying from deep blue in alkaline or weakly acidic media to yellow–orange in stronger acidic conditions. It is redox-sensitive, changing from reduced yellow to oxidized red forms in solution.

This synthetic colourant has been used in flavoured drinks, decorations and coatings for pastry products, pastry and fine bakery products, confectionery including breath refreshing and chewing gum, flavoured fermented milk products, edible cheese rinds, desserts, edible ices, several types of preserves of red fruits, seasonings, snacks, and alcoholic beverages [3].

It has been reported that after the oral administration of E131 in rats and dogs, low absorption prevails, with limited systemic availability and a mainly unchanged excretion in faeces [121]. Accordingly, the acceptable daily intake of 5 mg/kg_bw_ has been indicated [121]. Nevertheless, the consumption of this dye for people with allergic problems has been discouraged. There is also a possibility of the occurrence of potentially carcinogenic residues [16].

#### 2.2.10. Indigotine

**Figure 24:**
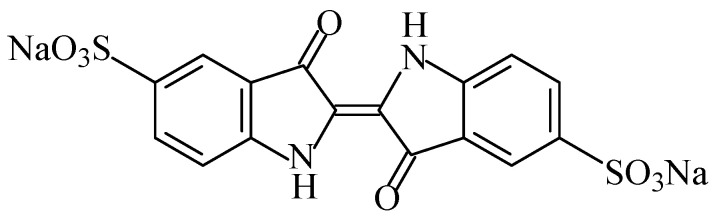
Structural formula of indigotine, or carmine-de-indigo (E132).

Indigotine, or “indigo carmine”, also called “Cl food blue 1”, is a food colouring (E132) of synthetic origin, occurring in the form of 5‚5′-indigodisulfonic acid sodium (C_16_H_8_Na_2_O_8_S_2_), or as calcium and potassium salts (Figure 24). Indigotine is a 0.2% aqueous solution, and is blue at pH 11.4 and yellow at 13.0. It is very sensitive to light and oxidizing agents such as bleach. This food colour is present in the form of dark blue powders or granules which are sparingly soluble in ethanol [7,122].

E132 has been used in flavoured drinks, toppings and decorations for pastry products, confectionery including breath refreshing and chewing gum, flavoured fermented milk products, edible cheese rinds, seasonings, desserts, edible ices, snacks and alcoholic beverages.

An acceptable daily intake of 5 mg/kg_bw_ has been indicated [123], but no adverse effects have been shown in subacute, chronic, reproduction or developmental toxicity studies, nor have modifications of haematological and biological parameters in chronic toxicity studies been identified at lower doses than 500 mg/kg_bw_ [123]. Moreover, side effects on human health related to allergy problems, as well as the possible existence of residues of potentially carcinogenic substances, have been reported [16]. Besides this, the simultaneous consumption of delicatessen products where sodium nitrite (E250) is used is not recommend (Voss, 2011). Inetianbor et al. [18] further reported, as possible negative health effects, nausea, vomiting, skin rashes, and brain tumours in animals.

#### 2.2.11. Brilliant Blue FCF 

**Figure 25:**
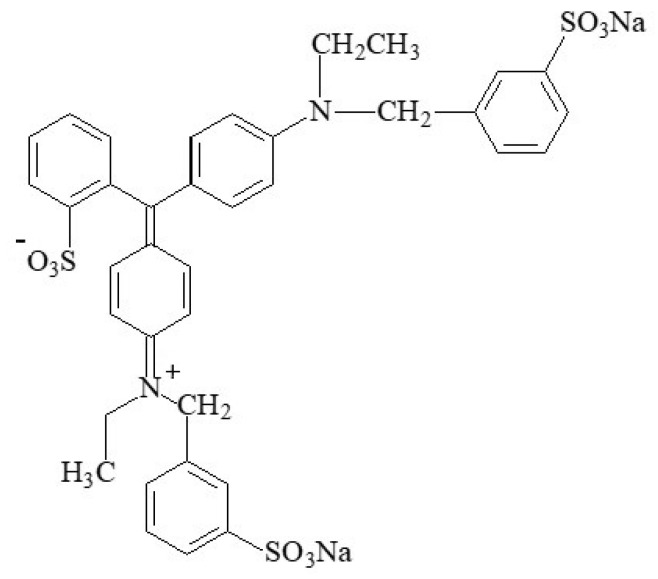
Structural formula of brilliant blue FCF (E133).

Brilliant blue FCF (E133), also known as “Cl food blue 2”, is a triarylmethane dye (C_16_H_8_N_2_Na_2_O_8_S_2_). It is a blue-coloured synthetic dye which is very sensitive to oxidizing agents (bleach) and light [7], and is soluble in water and glycerol, but slightly soluble in ethanol [124]. It is a sodium, calcium or potassium salt (Figure 25).

Brilliant blue FCF, which can be presented in the form of reddish-blue powders or granules, is used in flavoured drinks, flavoured fermented milk products, edible cheese rinds, seasonings, desserts, edible ices, confectionery including breath refreshing and chewing gum, preserves of red fruits, processed mushy and garden peas (canned), snacks, and alcoholic beverages [3].

Only about 5% of the ingested brilliant blue FCF is absorbed from the gastrointestinal tract, and the remainder occurs in the feces. However, it can be absorbed directly into the bloodstream through the tongue or shaved skin [125]. In this context, an acceptable daily intake of 6 mg/kg_bw_ has been reported [126]. Concerning the side effects on human health, [16] reported that, in high doses, it could cause deposits in the kidneys and lymphatic vessels, and that residues of potentially carcinogenic substances can also occur. Inetianbor et al. [18] further highlighted, as possible negative health effects, hyperactivity and skin rashes, DNA damage and tumours (found in animals).

#### 2.2.12. Green S 

**Figure 26:**
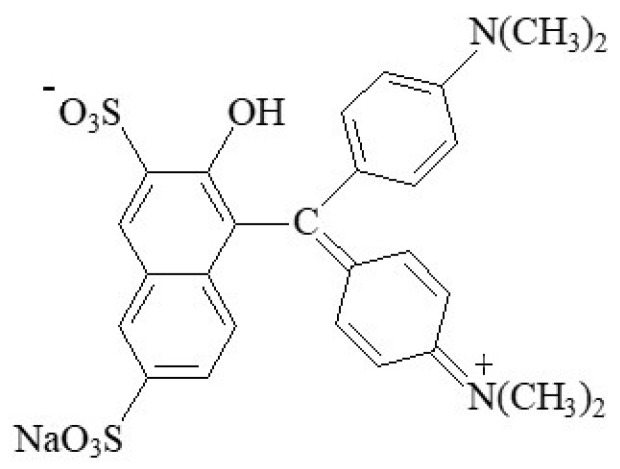
Structural formula of green S (E142).

Green S, “brilliant green BS”, or “Cl food green 4” is a green synthetic coal tar triarylmethane food colouring (E142) in the form of sodium (Figure 26) (C_27_H_25_N_2_NaO_7_S_2_), potassium or calcium salts [7]. This dye is soluble in water but only slightly soluble in ethanol [127], and comes in the form of powders or granules, in a dark-green to dark-blue colour. 

Green S is used in syrups of candied fruits, fruits and vegetables, marmalades, jams and jellies, flavoured drinks, confectionery including breath refreshing and chewing gum, pastry and fine bakery products, decorations and coatings, desserts, flavoured fermented milk products, edible cheese rinds, ice creams, soups, sauces, precooked crustaceans, fish paste and crustacean paste, processed mushy and garden peas (canned), seasonings and alcoholic beverages [3].

An acceptable daily intake of 5 mg/kg_bw_ has been indicated [128]. Harmful side effects on human health have been described when consumed in very high doses, and there is a possibility that there may be residues of potentially carcinogenic substances [16]. However, the data on the absorption, distribution, metabolism and excretion of green S show that it is poorly absorbed, and is mainly excreted unchanged in faeces [128].

#### 2.2.13. Brilliant Black PN

**Figure 27:**
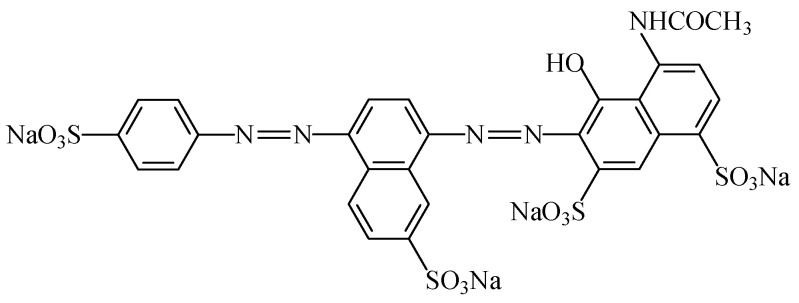
Structural formula of brilliant black PN (E151).

Brilliant black PN, also referred to as “CI food black 1”, is an artificial black diazo food colouring (E151) in the form of sodium (C_28_H_17_N_5_Na_4_O_14_S_4_), potassium or calcium salts (Figure 27). It occurs in the form of black powder or granules, being soluble in water but sparingly soluble in ethanol, and giving a black colour to foodstuffs [7,129]. 

This food colouring is used in confectionery including breath refreshing and chewing gum, pastry and fine bakery products, flavoured fermented milk products, flavoured drinks, desserts, edible ices, edible cheese rinds, fish roe, seasonings, soups, cheese rinds, edible casings, fish paste and crustacean paste, precooked crustaceans, smoked fish, appetizers and alcoholic beverages [3,8].

An acceptable daily intake of 1 mg/kg_bw_ has been suggested [129]. Side effects to human health similar to those indicated for other azo food colours have been described [16]. Although, after azo’s reduction in the gastrointestinal tract, free sulphonated aromatic amines may reach the systemic circulation, concerns to genotoxicity prevail [130].

#### 2.2.14. Brown HT

**Figure 28:**
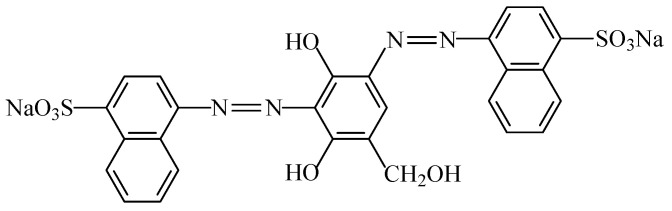
Structural formula of brown HT (E155).

Giving a reddish-brown colour, the food colouring brown HT (E155), also known as “Cl food brown 3”, is an azo food dye in the form of sodium (C_27_H_18_N_4_Na_2_O_9_S_2_), calcium or potassium salts (Figure 28), being a brown powder or granules which are soluble in water and insoluble in ethanol [7,131].

Brown HT can be used in most food products, namely in pastry and fine bakery products, confectionery including breath refreshing and chewing gum, flavoured fermented milk products, desserts, edible cheese rind, ice creams, smoked fish, soups, appetizers, unprocessed meat, precooked crustaceans, seasonings, sauces, flavoured drinks, and alcoholic beverages [3]. 

An acceptable daily intake of 1.5 mg/kg_bw_ has been suggested [132], with some side health effects being reported by Voss [16], namely allergic reactions, mainly in asthmatics and people with an intolerance to acetylsalicylic acid, or those who suffer from skin allergies. This additive is suspected of hyperkinesia, and may have residues of potentially carcinogenic substances. When consumed in high doses, experiments with rats revealed deposits in the kidneys and lymph nodes [16]. However, carcinogenic effects were not detected in long-term toxicity tests with rats and mice [132].

#### 2.2.15. Litholrubine BK

**Figure 29:**
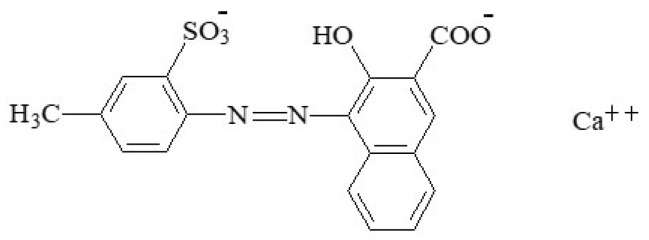
Structural formula of litholrubine BK (E180).

Litholrubine BK (E180), also known as “CI pigment red 57, “rubin pigment” or “carmine 6B” (Figure 29), is an azo food colouring of synthetic origin which is only used to give a red colour to edible cheese rinds. It is a red powder which is slightly soluble in hot water (90 °C) but insoluble in cold water and in ethanol [3,7,133]. 

Some side effects on human health, arising from the consumption of this dye are similar to those of other azo dyes, (i.e, it is an allergizing substance). Additionally, it is suspected of hyperkinesia, and there is the possibility of having residues of potentially carcinogenic substances [16]. However, there is not a significant safety concern for humans in edible cheese rinds [134,135].

## 3. Conclusions

The increasing variety of food production, which is associated with the diversification of technological developments and changes in consumers’ nutrition habits, has increased the number of processed foods. In this context, the colouring additives applied to food products play an important role in consumers’ preferences. However, their preferences for naturally derived colourants—which are closely associated to the image of healthy, safe and good-quality products—sharply increased, as a large number of synthetic dyes have recognized side effects on human health. Thus, considering the challenge imposed to the food industries, in order to surpass the technological limitations imposed by richly coloured pigments due to their properties/abilities (i.e., the chemical stability of naturally derived food pigments that is affected pH, temperature, light, oxygen, solvents, enzymes, proteins and metallic ions), alternatives must be developed. Accordingly, to overcome these constraints, new and specific sources and procedures must be recognized in order to improve not only the stability of food colouring additives and the final attractiveness of enriched-foodstuffs during the processing and storage practices but also their safety. Indeed, the use of food colourants must be performed within the limits and to fulfill a certain function (within the legal framework), whilst facing the whole impact at short and long terms. Besides this, although consumers have a great perception of the undesirable and harmful effects triggered by synthetic colourants, the strong advantage of natural pigments still requires a conclusive demonstration of health-improving/maintenance effects.

## Figures and Tables

**Table 1:** Chlorophylls, chlorophyllins and their cupric complexes used as food colours.

Additive Code	Designation	Description (Colour and Appearance)
E 140(i)	Chlorophylls	Waxy solid ranging in colour from olive green to dark green depending on the content of coordinated magnesium
E 140(ii)	Chlorophyllins	Dark green to blue/black powder
E 141 (i)	Copper complexes of chlorophylls	Waxy solid ranging in colour from blue green to dark green depending on the source material
E 141(ii)	Copper complexes of chlorophyllins	Dark green to blue/black powder

**Table 2:** Caramels used as the food colour E150 and their preparation.

Additive Code	Designation	Preparation
E 150a	Plain Caramel, Caustic caramel	Controlled heating of carbohydrates with or without addition of acids or bases. No sulphite or ammonium compounds are used.
E 150b	Caustic Sulphite Caramel	Controlled heating of carbohydrates with or without addition of acids or bases, in the presence of sulphite compounds ^(^^a)^. No ammonium compounds are used.
E 150c	Ammonia Caramel	Controlled heating of carbohydrates with or without addition of acids or bases, in the presence of ammonium compounds ^(^^b)^. No sulphite compounds are used.
E 150d	Sulphite Ammonia Caramel	Controlled heating of carbohydrates with or without addition of acids or bases, in the presence of both sulphite and ammonium compounds ^(^^c)^.

(a) The sulphite compounds are sulphurous acid, potassium sulphite, potassium bisulphite, sodium sulphite and sodium bisulphite. (b) The ammonium compounds are ammonium hydroxide, ammonium carbonate, ammonium hydrogen carbonate and ammonium phosphate. (c) The sulphite ammonium compounds are sulphurous acid, potassium sulphite, potassium bisulphite, sodium sulphite, sodium bisulphite, ammonium hydroxide, ammonium carbonate, ammonium hydrogen carbonate, ammonium phosphate, ammonium sulphate, ammonium sulphite and ammonium hydrogen sulphite.

**Table 3:** Iron oxides and hydroxides used as colours.

Designation	Chemical Name	Chemical Formula
Iron oxide Black or “Cl pigment black 11”	Ferroso and ferric oxide, iron (II, III) oxide	FeO·F_2_O_3_
Iron oxide red or Cl pigment red 101 and 102	Anhydrous ferric oxide, anhydrous iron (III) oxide	Fe_2_O_3_
Iron oxide yellow or “Cl pigment yellow 42 and 43	Hydrated ferric oxide, hydrated iron (III) oxide	FeO(OH)·xH_2_O
